# Knowledge, attitudes, and practices related to COVID-19 in Indonesia: A post delta variant wave cross-sectional study

**DOI:** 10.3389/fpubh.2023.1072196

**Published:** 2023-04-13

**Authors:** Firdaus Hafidz, Insan Rekso Adiwibowo, Gilbert Renardi Kusila, Ayunda Oktavia, Benjamin Saut, Citra Jaya, Dedy Revelino Siregar, Erzan Dhanalvin, Indira Tania, Johana Johana, Mahlil Ruby, Wan Aisyiah Baros

**Affiliations:** ^1^Department of Health Policy and Management, Faculty of Medicine, Public Health, and Nursing, Universitas Gadjah Mada, Yogyakarta, Indonesia; ^2^Center for Health Policy and Management, Universitas Gadjah Mada, Yogyakarta, Indonesia; ^3^Badan Penyelenggara Jaminan Sosial Kesehatan, Jakarta, Indonesia

**Keywords:** COVID-19, knowledge, attitude, practices, vaccination, health seeking behavior, Indonesia

## Abstract

**Introduction:**

Public information and regulations related to the coronavirus disease 2019 (COVID-19) have been widely published and continuously changed. The Indonesian government has responded to the emerging evidence by regularly updating its unprecedented and preventive measures against the transmission of COVID-19 to the public. It is important to understand how the public responded to these updates. This study aimed to investigate the knowledge, attitudes, and practices of Indonesians toward COVID-19 after the emergence of the delta variant wave.

**Methods:**

A cross-sectional study was conducted among the adult population of non-healthcare workers in Indonesia through an online questionnaire using the SurveyMonkey platform. A total of 1,859 respondents completed this survey from September to October 2021. The knowledge, attitudes, and practices data were analyzed descriptively to find their frequency and percentage. A multivariate analysis was conducted to confirm the factors affecting the respondents' knowledge, attitudes, and practices with a *p*-value of <0.05 set as significant.

**Results:**

Being female, having a higher education level, and having a higher frequency of access to COVID-19 news showed significant impacts on knowledge, attitudes, and practices (*p*<0.001). Older age stratification influenced the knowledge level (*p*<0.05) but had no significant effect on people's attitudes and practices toward COVID-19. Respondents' perceived probability of being exposed to COVID-19 (*p* < 0.05) and their COVID-19 infection frequency (*p* < 0.001) significantly influenced their knowledge. Household income and respondents' knowledge significantly affected their attitudes toward COVID-19. Furthermore, only their attitudes had a significant impact on the respondents' practices. Perceived severity, perceived susceptibility, and vaccination status did not significantly influence their knowledge, attitudes, and practices (*p* > 0.05).

**Conclusion:**

After more than a year of the COVID-19 pandemic, Indonesians maintain their high level of knowledge, attitudes, and practices. COVID-19 disinformation must be combatted by strengthening authorized media, empowering communities, and improving governance among institutions during and post-pandemic.

## Background

Since the World Health Organization (WHO) declared that coronavirus disease 2019 (COVID-19) is a global pandemic, governments around the globe have been developing and implementing response plans to contain the spread of the virus (1). As more studies emerge, more evidence on the disease becomes apparent and in response, public health measurement efforts are also evolving. One of the most prominent examples is the update on the public use of masks and COVID-19 diagnosis assessment. During the early stage of the pandemic, the WHO suggested healthy people should refrain from wearing face masks. The information was then revised when the WHO required everybody to wear face masks regardless of their health status (2, 3). Similarly, the latest WHO guidance has also allowed the SARS-CoV-2 antigen rapid testing to be performed and interpreted by individuals without healthcare workers' supervision (4).

In Indonesia, a circular letter from the Ministry of Health to the regional governments was the first piece of policy delivered on COVID-19 public health measurements. This letter also mentioned the penalties for disobedience (5). To date, Indonesia has never implemented a full lockdown policy. However, in the very early phase of the pandemic, government regulation was launched to promote large-scale social restrictions (6). Nevertheless, this restriction policy was never mandatory since it purely relied on each district-provincial government's proposal that explained the regional urgency and readiness to implement the restriction. Such restrictions must be approved by the Ministry of Home Affairs (MoHA) (6). Over time, this regulation was annulled and replaced by the MoHA orders that imposed community–activities–restriction–enforcement, which divided municipalities into four different restriction levels mainly based on their COVID-19 confirmed cases. The implementation of these levels differs depending on the regional success or failure to overcome the incidence; hence, these lists of “red zone” municipalities were updated regularly in the MoHA orders (7, 8).

However, all of these efforts are useless without adequate support and adherence from society (9). The adherence to these preventive practices is likely to be influenced by the public's knowledge and attitudes toward COVID-19, especially when the COVID-19 information comes from public figures including health workers (10, 11). Both developed and low-to-middle-income countries showed a similar pattern, which explained that knowledge is essential to establish appropriate awareness, perceptions, attitudes, and practices of the pandemic. Other factors also determined these findings such as gender, age, urban–rural disparity, economic status, and education level (12–14). Similarly, a meta-analysis study concerning public responses to influenza showed that both the knowledge gap in the population and the undefined standard of what is considered a sufficient protection effort showed insignificant impact on preventive interventions (15).

The sources of the information (government and social media) were found to affect public knowledge, trust, and adherence to COVID-19 prevention policies (16). This is a challenging issue since any circulated narratives including those coming from untrusted sources mixed with pre-existing cultural beliefs and myths could be misperceived as evidence-based science and lead to incorrect health practices (13, 17, 18). Furthermore, media consumption during this pandemic has increased by >50% on multiple platforms with a relatively larger percentage of people who were likely to maintain the high rate of their current use (19). Assessing public knowledge, perception, attitudes, and practices related to COVID-19 is becoming crucial to plan for future health promotions, campaigns, approaches, and community empowerment programs to strengthen the ongoing COVID-19 countermeasures.

This study investigates the knowledge, attitudes, and practices (KAP) of Indonesians toward COVID-19 during the height of the pandemic, which was caused mainly by the delta variant between June and September 2021. During that period, the daily COVID-19 confirmed cases hit a record high with 56,757 new cases on 15 July 2021 (update: this was surpassed during the Omicron wave with 57,049 daily new cases in February 2022) and the COVID-19-related deaths skyrocketed up 348% in just 1 month (20). This wave started after a long national holiday and led to massive mobility of people, which was followed by premature COVID-19 measurement loosening and low compliance from the citizens (21). During this period, Indonesia experienced a national-scale oxygen shortage. In one provincial central hospital, half of the COVID-19 patients died due to central oxygen running out. Furthermore, the bed occupation rate surpassed 80% in many provinces, forcing them to utilize the parking lot and emergency tents as COVID-19 wards (21, 22). This period was chosen to investigate how the healthcare system collapsed due to extremely high infection rate and whether the death toll may affect or change people's understanding, believes, and habits. Furthermore, we assumed that a year of the pandemic had given the public enough time to access COVID-19 information. Therefore, there would be minimal bias resulting from guessing the answers to the given questions.

## Methods

### Research design and sample

This study was conducted using a cross-sectional design. The demographic data, which also included their socioeconomic background, knowledge, attitudes, practices, and other COVID-19-related information, were collected using an online survey form through the SurveyMonkey platform (www.surveymonkey.com). The online survey was distributed by the Social Security Administrative Body of Health (*BPJS Kesehatan*). The link to the online questionnaire was delivered through the *BPJS Kesehatan* district offices and its mobile application (Mobile JKN) to 33 provinces in Indonesia (note: since the second half of the year 2022, there are now 37 provinces in Indonesia). Data were collected from 23 September 2021 to 7 October 2021. We did not give any compensation to the respondents. This survey used convenience sampling methods. To be involved in this study, participants should be Indonesian citizens, at least 18 years old or older, and not working as healthcare workers (doctors, nurses, midwives, etc.). The sample size was estimated using an online sample calculator named Raosoft. For applying a 95% confidence level, a 3% margin of error, a 50% response distribution, reliability to represent the 272,229,372 Indonesian population, and a minimum sample of 1,068 were required. This study collected data from a total of 1,859 respondents who completed the survey. This study obtained ethical clearance from the Medical and Health Research Ethics Committee of the Faculty of Medicine, Public Health and Nursing, Universitas Gadjah Mada, Yogyakarta, Indonesia, with approval no. KE/FK/0945/EC/2021.

### Instrument development

The questionnaire was separated into five sections and used the standardized Indonesian language. Before the respondents were directed to the questions, they would be introduced to the information regarding the study. This part mentioned the organizers, goals of the study, respondents' filling instructions, the time needed to complete the survey, data analysis, data utilization, contact person, and the confidentiality of the data. Once the respondents had read the information, the next step asked for their consent to participate. Only those who fully understood and voluntarily agreed could move to the next sections. Those who did not agree would automatically end the survey.

The first section of the questionnaire was the demographic and socioeconomic section which contained questions about age, gender, education, occupation, number of household members, marital status, status in the family (main breadwinner, husband, wife, child, and elderly), province of origin, monthly household income, monthly COVID-19 related expenses, and health insurance (BPJS Kesehatan) class of service. The analysis in this article only focused on age, gender, education, and monthly household income.

The next three parts assessed the respondents' KAP toward COVID-19. The assessment was adapted from the Centers for Disease Control and Prevention (CDC) and WHO questionnaire (23, 24). The questions were then adjusted with the most updated COVID-19 information attached to Indonesia's Ministry of Health website that could be publicly accessed (25). To assess the respondents' knowledge, a total of 20 statements were given. The statements were classified into five categories: COVID-19 transmission, preventive measures, symptoms, treatments, and risk factors. The respondents should determine whether the statements were “true” or “false”. For each correct answer, they earned five points, hence the maximum score would be 100. After they completed the survey, their knowledge score would appear, their mistakes were shown, and correct answers were explained.

For the attitude assessment, the respondents were asked about their agreement to seven statements using a 5-point Likert scale (strongly disagree, disagree, neutral, agree, and strongly agree). Similarly, they next responded to six statements related to COVID-19 practices based on their frequency in conducting preventive activities (never, seldom, often, and always). Specifically, the respondents answered the statements related to their practices over the past 3 months. The duration of 3 months before the survey was selected based on the period of the emerging delta wave until its declining trend.

The last part of the survey consisted of some additional information. The respondents were required to score from one (impossible) to nine (most possible) about their risk of being exposed to COVID-19 based on their daily activities and habits (perceived probability), how they could possibly get infected and transmit the virus (perceived susceptibility), and the severity if they were infected by COVID-19 (perceived severity) (23). These three perceptions were included to give a better understanding of the knowledge and behavioral insight on COVID-19, which was adapted from the WHO Regional Office for Europe's guideline (23, 26). Their history of contracting COVID-19 was classified as follows: have ever been confirmed positive for COVID-19, have been suspected but never been tested, have been suspected but tested negative for COVID-19, have never been confirmed or suspected, and have unknown status. Their COVID-19 vaccination status was also included concerning whether they had received the first or second vaccine or were not yet vaccinated. The frequency of accessing COVID-19-related news from any mass or social media (but not in private or group conversations) was classified as always, often, seldom, or never.

The instrument was first distributed to 50 people to gain an external evaluation. These evaluators were BPJS *Kesehatan* employees, public health postgraduate students of Universitas Gadjah Mada, medical doctors from various public hospitals, and members of non-governmental organizations involved in COVID-19-related health campaigns. Some sentences that indicated multi-interpretations were revised. Moreover, the sentences that included local-dialect terms were fixed to the formal and standardized Indonesian language. No questions were annulled during the process. The ineffective phrases were compressed, together with the type of the form, resulting in a reduction of 1 min for the survey to be accomplished in only 7 min. Then, it was redistributed to 100 people with non-academic non-health-related backgrounds from different regional dialect origins as a pilot study to receive further evaluation and to conduct validity and reliability testing. Cronbach's alpha reliability testing had values of >0.60, which made the instrument reliable and acceptable.

### Data analysis

A descriptive analysis was used to show the frequency and percentage of the participants' characteristics and the statements in the KAP sections which consisted of categorical data. For perceived probability, susceptibility, and severity, the results were tabulated as averages and standard deviation (SD), while the monthly income was written as the median and interquartile range (IQR).

This study then employed multivariate analyses using logistic regression to portray the distinct effects of age group, gender, education level, vaccination status, COVID-19 history, and accessing COVID-19 news on the respondents' KAP. For age, the respondents were grouped into 18–29, 30–39, 40–49, and 50–59 years (24). For the education level, the analysis was done by categorizing the data into “high school and below” and “bachelor and above” (24). The linear regression was conducted to analyze the association of average household income, perceived probability, perceived susceptibility, and perceived severity on the KAP (23).

Furthermore, testing was also done to see the influence of knowledge (independent variable) on attitude and practices (dependent variables), and also the impact of attitude (independent variable) on practices (dependent variable). Both were tested by regression analysis. The knowledge level was determined by participants' scores from their correct answers. The Likert scale for the attitude was rearranged for the statistical analysis. A score of 5 was given for the most positive attitude to COVID-19 measurements. Meanwhile, the most unsupportive attitude would be scored 1. Similarly, the COVID-19-related practices were scored from 1 to 4, where 4 shows the frequency that was in accordance with the regulations (23, 24). All analyses were performed with a 95% confidence interval (CI) and a significance set as a *p*-value of <0.05 using R version 4.0.4 (R Foundation for Statistical Computing, Vienna, Austria).

## Results

A total of 1,859 respondents completed the questionnaire. Table 1 shows the social and demographic characteristics of the study participants. The respondents were predominantly women (55%), with the majority between 30 and 39 years (44%). They were mostly married (70%) and had received college degrees (77%). Information about COVID-19-related news was also collected, where most respondents seldom accessed that news (46.8%). Moreover, respondents showed a relatively countable percentage of history of COVID-19 confirmed cases (32.6%). Approximately 11.1% of them suspected they had the virus, and only 48.7% of them had never been diagnosed with or suspected of COVID-19. Almost all respondents also had been vaccinated (93.3%) with the full dosage (second dose). In addition, it was shown that the respondents gave an average score of 5.8 for the possibility of being infected by COVID-19 and a neutral position in perceived susceptibility score (4.9). However, they believed that they were less likely to develop a severe COVID-19 infection (3.7). From the total of 20 questions related to COVID-19, the mean score from the respondents' correct responses was 86.0. The mean for the individual's attitude and practices score were 4.43 and 3.33, respectively.

**Table 1:** Social and demographic characteristics of participants.

**Characteristics**	***N* = 1,859**
**Gender**	
Male	832 (45%)
Female	1,027 (55%)
**Age group**	
18–29	766 (41%)
30–39	822 (44%)
40–49	229 (12%)
50–59	42 (2.3%)
≥60	0 (0%)
**Education level**	
Junior high school or equal	6 (0.3%)
Senior high school or equal	275 (15%)
Diploma and undergraduate	1,433 (77%)
Postgraduate	144 (7.7%)
**Accessing COVID-19 news**	
Always	131 (7.0%)
Often	652 (35.1%)
Seldom	870 (46.8%)
Never	206 (11.1%)
**COVID-19 status**	
Ever confirmed	606 (32.6%)
Suspected, not tested	57 (3.1%)
Suspected, tested negative	148 (8.0%)
Never been confirmed or suspected	904 (48.7%)
Do not know	144 (7.8%)
**Vaccination Status**	
Not yet	58 (3.2%)
1st dose	67 (3.6%)
2nd dose	1,732 (93.3%)
**Knowledge, attitude, practice score**	
Knowledge^*^	86.0 (0.71; 0.90)
Attitude^*^	4.43 (4.00; 4.71)
Practices^*^	3.33 (3.17; 3.50)
**Household income**	
Monthly Household Income^*^ (million rupiah)	10.0 (5.00; 15.0)
**Perceived risk of COVID-19**	
Perceived Probability^**^	5.8 ± 2.6
Perceived Susceptibility^**^	4.9 ± 2.4
Perceived Severity^**^	3.7 ± 2.5

* Mean (CI).

** Average score ± standard deviation.

From the five subthemes asked, attached in Table 2, the statements about COVID-19 preventive measures reached the highest number of correct responses. Only one question about the type of mask for the public had below 90% correct responses. Although the respondents understood its preventive measures, only a few could elaborate on how COVID-19 was transmitted. More than a quarter of the respondents gave false responses to the COVID-19 transmission statements. Furthermore, we also found that the public still could not differentiate the symptoms of COVID-19 from influenza or the common cold, as shown in Table 2. Approximately 67% of the respondents agreed that a runny nose and sneezing were common symptoms among those infected by COVID-19. Moreover, their knowledge regarding the COVID-19 treatment and its risk factors showed some variations. The public understood the need for isolation as soon as COVID-19 was confirmed by the diagnostic test, however, their comprehension of the medication was still lacking. They believed that the elderly and those with chronic illness, but not pregnant women, have a higher risk of developing a more severe infection.

**Table 2:** Knowledge response to COVID-19 in Indonesia.

**Statements**	**Correct responses**	**False responses**
	***N* (%)**	***N* (%)**
**Transmission**		
Exposure to droplets (splashes of water) that produced when someone talks, coughs, or sneezes can transmit the virus.	1,354 (73%)	505 (27%)
The droplets can be transmitted in < 2 meters distance.	1,276 (69%)	583 (31%)
Touching an object that is occupied by the virus and then touching the face area can increase the risk of infection.	1,342 (72%)	517 (28%)
**Preventive measures**		
After being in a public place, coughing, or sneezing, a person should wash their hands with water and soap or hand sanitizer for at least 20 seconds.	1,764 (95%)	95 (5.1%)
It's best to avoid touching your eyes, nose and mouth with unwashed hands.	1,813 (98%)	46 (2.5%)
The general public can use cloth masks or medical masks to prevent COVID-19.	1,654 (89%)	205 (11%)
A person only uses a mask if he is infected with the virus or is treating someone with COVID-19 symptoms.	1,666 (90%)	193 (10%)
Eating a healthy diet and drinking lots of water can boost your immune system.	1,781 (96%)	78 (4.2%)
To prevent the transmission of COVID-19, one should avoid traveling to crowded places and taking public transportation.	1,717 (92%)	142 (7.6%)
**Symptoms**		
People infected by COVID-19 cannot spread the virus to others if they never develop a fever.	1,583 (85%)	276 (15%)
The main clinical symptoms of COVID-19 are fever, fatigue, dry cough, and shortness of breath.	1,709 (92%)	150 (8.1%)
Stuffy and runny noses, and sneezing are extremely common in people with COVID-19.	618 (33%)	1,241 (67%)
**Treatment**		
Giving antibiotics is an effective treatment for COVID-19.	721 (39%)	1,138 (61%)
Drinking eucalyptus oil is effective in dealing with COVID-19.	1,381 (74%)	478 (26%)
There is currently no effective treatment for COVID-19, although some early treatment could help the recovery.	1,325 (71%)	534 (29%)
Isolation and treatment of people with COVID-19 is an effective way to reduce the spread of COVID-19.	1,774 (95%)	85 (4.6%)
People who have a history of close contact with someone infected with COVID-19 should be immediately quarantined and observed for up to 14 days, even if they are asymptomatic.	1,682 (90%)	177 (9.5%)
**Risk factors**		
The elderly and people with chronic diseases, such as heart disease, asthma and diabetes, are at a higher risk of developing severe complications from COVID-19.	1,768 (95%)	91 (4.9%)
Pregnant women have the same risk to get COVID-19 infection as non-pregnant women.	965 (52%)	894 (48%)
Children and young people do not need to take measures to prevent the transmission of COVID-19.	1,772 (95%)	87 (4.7%)

Attitude aspects related to wearing masks, handwashing, and social distancing showed the highest percentage of strong agreement from the respondents as shown in Table 3. Nevertheless, fewer people gave similar responses to vaccination, social activities restriction, recommendation to stay at home, and penalties for health protocol violators.

**Table 3:** Attitude after the COVID-19 delta variant wave in Indonesia.

**Attitude**	**Agreement*****; N*** = **1,859**				
	**Strongly disagree**	**Disagree**	**Neutral**	**Agree**	**Strongly agree**
Keeping a distance from other people is important to prevent the spread of COVID-19	31 (1.7%)	7 (0.4%)	69 (3.7%)	603 (32%)	1,149 (62%)
Washing hands regularly can protect my family and me from COVID-19	27 (1.5%)	7 (0.4%)	54 (2.9%)	579 (31%)	1,191 (64%)
I should stay at home more to protect my family and myself from COVID-19	38 (2.1%)	96 (5.2)	235 (13%)	649 (35%)	835 (45%)
Using a mask correctly every time you go outside will reduce the risk of contracting COVID-19	29 (1.6%)	3 (0.2%)	40 (2.2%)	596 (32%)	1,187 (64%)
If most people have been vaccinated, the transmission of COVID-19 can be controlled	34 (1.8%)	79 (4.3%)	326 (18%)	794 (43%)	624 (34%)
I support the government's actions to carry out social and community activities restrictions to prevent the COVID-19 transmission	26 (1.4)	39 (2.1%)	256 (14%)	732 (39%)	803 (43%)
I believe that every health protocol violator must be dealt with firmly	38 (2.0%)	69 (3.7%)	417 (22%)	735 (40%)	599 (32%)

From the frequency of prevention practices, as shown in Table 4, it was found that wearing a face mask was the most common preventive action to be performed (98%), followed by handwashing (96%). Furthermore, 16.6% of the respondents still frequently attended face-to-face social activities such as weddings, funerals, and religious events. Nevertheless, recreational or shopping activities in relatively crowded places (malls, restaurants, and markets) were only frequently done by 8.8% of them, while 75% of them seldom did that. A total of 94% of the respondents admitted that they never or seldom traveled outside their hometown and only 6.7% of them were still actively visiting their non-close relatives during the pandemic.

**Table 4:** Practices after the COVID-19 delta variant wave in Indonesia.

**Practices**	**Frequency**			
	**Never**	**Seldom**	**Often**	**Always**
Wash hands with soap and water or hand sanitizer at least 20 seconds before eating, after being outside or in public places, and after coughing or sneezing	5 (0.3%)	70 (3.8%)	728 (39%)	1,056 (57%)
Use a mask when outside the house	2 (0.1%)	28 (1.5%)	226 (12%)	1,603 (86%)
Attending social events and social activities (weddings, funerals, recitations, social gatherings, eating together with friends, reunions, etc.)	339 (18%)	1,212 (65%)	223 (12%)	85 (4.6%)
Visiting crowded places (e.g. malls, markets, restaurants, discotheques, etc.)	305 (16%)	1,391 (75%)	143 (7.7%)	20 (1.1%)
Visiting other people's houses who are not close relatives (neighbors, friends, uncles, aunts, nephews, etc.)	552 (30%)	1,207 (65%)	89 (4.8%)	11 (0.6%)
Traveling out of town	718 (39%)	1,017 (55%)	106 (5.7%)	18 (1.0%)

Table 5 explains the factors that influence KAP. Age stratification influences the level of knowledge but had no significant effect on people's attitudes and practices toward COVID-19 (*p* < 0.05). Generally, the older the age of the respondents, the higher their level of knowledge about COVID-19, but in the age group of 50–59 years, the results were not significant anymore. Those aged 30–39 and 40–49 years had a higher level of knowledge about COVID-19 than those aged 18–29 years. Women have higher KAP toward COVID-19 than men, making a significant impact for gender on the level of knowledge (*p* < 0.05), attitude (*p* < 0.05), and practices (*p*<0.001) toward COVID-19.

**Table 5:** Factors affected the knowledge, attitude, and practices related to the post-COVID-19 delta wave situation in Indonesia.

**Characteristic**	**Knowledge**		**Attitude**		**Practices**	
	**ß**	**95% CI**	**ß**	**95% CI**	**ß**	**95% CI**
**Age**						
18–29	Ref	–	–	-	-	-
30–39	0.03	**0.01; 0.04** ^**^	−0.02	−0.09; 0.05	0.01	−0.03; 0.05
40–49	0.04	**0.01; 0.06** ^**^	0.04	−0.07; 0.15	0.05	−0.01; 0.10
50-59	0.04	−0.01; 0.09	−0.05	−0.28; 0.18	0.06	−0.06; 0.18
**Gender**						
Male	Ref	–	–	-	-	-
Female	0.02	**0.00;0.004** ^*^	0.11	**0.04; 0.18** ^*^	0.09	**0.05; 0.13** ^**^
**Education level**						
High school and below	Ref	–	–	-	-	-
Bachelor and above	0.09	**0.07; 0.12** ^**^	0.23	**0.12; 0.34** ^**^	−0.11	**-0.16;−0.05** ^**^
Average monthly income	0.00	0.00; 0.00	0.01	0.00; 0.04^*^	0.00	0.00; 0.00
**COVID-19 status**						
Ever confirmed	Ref	–	–	-	-	-
Suspected, not tested	−0.01	−0.06; 0.03	−0.06	−0.27;0.15	0.04	−0.07; 0.15
Suspected, tested negative	−0.01	−0.03; 0.02	−0.02	−0.15; 0.11	0.04	−0.02; 0.11
Never been confirmed or suspected	−0.03	**−0.05;** −0.02^**^	0.04	−0.03; 0.12	0.04	0.00; 0.08
Do not know	−0.06	**−0.09;** −0.03^**^	0.05	−0.09; 0.19	−0.07	−0.14; 0.01
**Perceived risk of COVID-19**						
Perceived Probability	0.01	**0.00; 0.01** ^*^	0.02	**0.00; 0.04** ^*^	−0.01	−0.02; 0.00
Perceived Severity	0.00	−0.01; 0.00	0.00	−0.02; 0.02	−0.01	−0.01; 0.00
Perceived Susceptibility	0.00	0.00; 0.01	−0.01	−0.02; 0.01	0.001	0.00;0.02
**Accessing COVID-19 news**						
Never	Ref	–	–	-	-	-
Seldom	0.03	**0.01; 0.06** ^*^	0.12	**0.01; 0.22** ^*^	0.00	−0.06; 0.05
Often	0.06	**0.03; 0.08** ^**^	0.20	**0.09; 0.31** ^**^	0.04	−0.02; 0.10
Always	0.05	**0.02; 0.08** ^*^	0.30	**0.16; 0.44** ^**^	0.13	**0.06; 0.20** ^**^
**Vaccination status**						
Not yet	Ref	–	–	-	-	-
1^st^ dose	−0.04	−0.09; 0.01	0.02	−0.20; 0.24	0.07	−0.04; 0.19
2^nd^ dose	−0.02	−0.05; 0.02	0.01	−0.16; 0.18	0.00	−0.08; 0.09
**Knowledge and attitude**						
Knowledge	–	–	0.82	**0.57; 1.1** ^**^	0.10	−0.03; 0.23
Attitude	–	–	–	-	0.10	**0.07; 0.13** ^**^

* p < 0.05;

** p < 0.001. The bold values were made to recognize the significant p-value quickly.

Furthermore, there was a significant difference (*p*<0.001) in the KAP of the people who accomplished higher education (college graduates) than the lower ones (high school graduates or below). In this study, average monthly income did not affect knowledge and practices, however, it showed positive and statistically significant results on people's attitudes toward COVID-19 (*p*<0.05).

The respondent's experience of being exposed to COVID-19 was classified into five categories: (1) has been confirmed positive for COVID-19; (2) has been suspected but not tested; (3) has been suspected but tested negative for COVID-19; (4) has never been confirmed or suspected; and (5) does not know their COVID-19 status history. Statistically, there was no significant effect of a history of being suspected of COVID-19 on KAP in dealing with the COVID-19 pandemic. The level of knowledge of those who had never been confirmed/suspected and the group who did not know their COVID-19 history showed a significant difference (*p* < 0.001), but there were no significant differences in attitudes and practices compared to the confirmed positive group for COVID-19. The effect was negative, which means that those two groups have lower knowledge than the confirmed group.

Perceived probability had a significant effect on the respondent's level of knowledge (*p* < 0.05) and attitudes (*p* < 0.05) but not on their practices toward COVID-19. Meanwhile, perceived severity and perceived susceptibility showed no significant effect on KAP (*p* > 0.05). Similarly, vaccination status also did not significantly influence the level of KAP (*p* > 0.05).

The level of public awareness influences their knowledge and attitudes toward COVID-19. People who admitted to always catching up with COVID-19 news had significantly different KAP than those who had lower levels of accessing the news (*p* < 0.05). The level of knowledge significantly affects a person's attitude toward COVID-19 (*p* < 0.001), but not their practices. On the contrary, the respondents' attitudes showed a significant effect on their practices (*p* < 0.001).

## Discussion

To the best of our knowledge, this study is the first study to investigate the KAP related to COVID-19 of Indonesia's citizens after the lethal delta variant wave. Some similar studies were conducted in the early phase of the pandemic, however, those studies only explored the KAP on specific medical-related occupations and excluded those with a history of COVID-19 positive test results (27–29). Meanwhile, a previous study in the Indonesian adult population only highlighted hand hygiene, although it could better elaborate on the perceptions and practices in various situations where hand hygiene is performed (30). In this study, we explored more of the preventive measures and did not specifically separate the timing of hand hygiene. Nevertheless, 57% of our respondents answered that they always performed hand hygiene before eating, after being outside or in public places, and after coughing or sneezing, which is a relatively similar percentage compared to a previous study in this pandemic (30).

The respondents in this study unexpectedly reached a high score for their COVID-19 knowledge with an average score of 86. These scores were also higher in comparison with a study in 2020 among 1,167 Indonesian citizens, whose median score was 24 (31). Among the lowest correct answers were the questions related to the COVID-19 symptoms and the medication.

Although identifying flu-like symptoms was less, many respondents believed them to be dominant COVID-19 symptoms (32). Those flu-like symptoms were often misconstrued due to their similarity with cough as a body protective response. Remarkably, somebody's protective reflex may be perceived as a threat during the pandemic due to the increasing fear and stigma of being sick (32). It was assumed that the excessive use of COVID-19 diagnostic tools without medical indication in Indonesia could deceive people's perceptions regarding COVID-19 symptoms. During the pandemic, Indonesia's government regulated the use of these diagnostic tools for medical and travel purposes. However, many institutions perform the test for other purposes, especially for face-to-face gatherings, hence, the COVID-19 tests are often independently ordered by event organizers, public spaces, working agencies, and families as a self-regulated meeting requirement. It was discovered that a sense of personal responsibility and psychosocial reason often led to personal requests for a diagnostic test (33). However, this was not followed by the full knowledge of the disease, resulting in more unawareness of the bodily symptoms reinforced by the repeated use of unindicated tests (33).

Furthermore, the circulated information about what medication is best to treat COVID-19 also confused some people. Among all information, the use of eucalyptus oil was extremely widespread among Indonesian citizens and 26% of our respondents believed in its efficacy. Furthermore, although the third edition of the Indonesian COVID-19 Treatment Guideline allowed the use of antibiotics (Azithromycin) and additional phytopharmacy products, the public could not comprehend that no drug has ever been mentioned to be superior and efficacious enough (34). The unavailability of a specific robust medication to combat COVID-19 made people tend to rely on scientifically unproven medical treatments. This uncertainty is related to a favorable view of alternative medicines with boastful beneficial claims (17).

Our study suggested that although the respondents in this study had a relatively high education level, their understanding of medications was still inadequate. Our respondents may again give a simple illustration where 61% of them agreed that COVID-19 should be treated with antibiotics. This probably was not limited to COVID-19 alone, but probably any other disease medication information. This should be further evaluated, especially since the pandemic has accelerated the utilization of e-commerce for online medication self-purchasing (35). When the details about indications, contraindications, and further safety information were not well-informed, potential harm may be experienced by the public, including further antibiotic resistance. To overcome this, many institutions should collaborate in educating society about medication utilization, followed by stricter regulations. Findings in a tuberculosis-related study suggested the importance of community pharmacists in detection, drug consultation, and treatment provision services (36). Just as our study also showed the participants' knowledge of medication therapy was inferior, pharmacists who understand the local wisdom, availability of essential drugs, and society's medication patterns might be empowered to bridge this important issue.

Interestingly, the question “Pregnant women have the same risk of getting COVID-19 infection as non-pregnant women” was inorrectly answered by 48% of respondents. This can be attributed to vague health promotion messages in which “pregnant women have a higher risk for severe COVID-19 infection” are often simplified only as pregnant women are high-risk people. This vague message made the differentiation between a higher risk of infection and a higher risk of severity unclear among the general public. However, to what extent this understanding may lead to the increasing vulnerability of pregnant women is still unknown.

Gender, educational level, and always accessing COVID-19-related news were three factors associated with the KAP of this study's participants. Older age was only significantly correlated to better knowledge but did not affect attitude and practices. These findings were rather different from other studies.

Studies among Pakistani health workers and the Bangladeshi population showed that men and women performed the knowledge test similarly. However, age significantly affected their knowledge score (37, 38). Bangladeshi women had significantly better attitudes and practices to combat the pandemic than their male counterparts. This also included the concern for children's outdoor activities (38). The duration of formal education was surprisingly insignificant with KAP in Bangladesh. They used 12 years of formal education as a cutoff, which is equal to graduating from high school in Indonesia (38).

On the contrary, the age among the Ethiopian population significantly impacted the attitude but had no significant association with knowledge and practices toward COVID-19 (39). These findings may vary due to the subjects' characteristics. Older ages may have better comprehension resulting in a higher level of knowledge. However, they had more difficulty creating novel perceptions and practices toward change (40).

Another study mentioned that women, older age, and higher income were significantly associated with knowledge and preventive practices in the Malaysian population. These groups had significant confidence that COVID-19 could be successfully controlled (41). In accordance with the Malaysian study, our respondents showed an association between higher household income and a better attitude. Still, the monthly household income showed no significant effect on our participants' knowledge and practices. In addition, better COVID-19 knowledge was found among the higher socioeconomic class in Iranian adults and was also determined by family income. Nevertheless, coming from this group did not give a significant improvement on attitude and practices (42). Their study also found that having a college degree improved COVID-19-related attitudes (42). A study in India stated that both economic status and education level were the significant determinants for overall COVID-19 KAP (43).

Adopting health measurement practices and experiencing economic instability during the pandemic would also contribute to individuals' and families' ability to purchase health-related items or services. In particular, there was an increasing demand for disposable items that may induce financial catastrophe (44). Therefore, the insignificant association between income and respondents' practices can occur from their purchase considerations.

Although almost all of the respondents were vaccinated with the second dose (93.3%), the attitude toward vaccines was relatively low compared to other attitudes, with 6.1% of them disagreeing that vaccination can control the disease. Remarkably, vaccination status showed no significant impact on the respondents' KAP. This finding was different compared to another study conducted in Bangladesh, which marked attitude and health measurement practices shifting among the vaccines. They found an increase in travel, face-to-face meetings, abandoning routine hand hygiene, and distance avoidance among the vaccines (45). Similarly, Corea et al. found that after taking the COVID-19 vaccines, people returned to doing more social activities. They also discovered that people who were still adhering to the health measurement behavior (social distancing, hand washing, and wearing a face mask) after receiving the COVID-19 vaccine had a tendency not to perceive COVID-19 as a serious illness (46).

We suggested that the low perceived severity, slightly higher perceived probability, and susceptibility given by our respondents could be because this study mostly gathered respondents who had received the second dose. This was in line with the Indonesian government campaign which stated that vaccination could mitigate the alarming COVID-19 symptoms, but not the risk of contracting the virus. This further expressed the significant association between perceived probability with the knowledge and attitude toward COVID-19, while none of the KAP were significantly influenced by the perceived severity and perceived susceptibility. A similar pattern was also found in Italy, where respondents with a higher perceived risk of contracting COVID-19 showed higher COVID-19-related news consumption (46). A study among the United Kingdom's university students also noted no significant association between these three perceptions and the KAP (23). However, they claimed that their insignificant results were caused by their study population which consisted of younger participants (23). A meta-analysis and systematic review conducted by Liang et al. discovered that perceived susceptibility had no significant impact on COVID-19 preventive behavior. Meanwhile, perceived severity led to significant initiatives to maintain social distancing (47).

Participants with no history of being diagnosed with or suspected of COVID-19 showed significantly lower knowledge compared to others. A study conducted among Egyptian medical students has observed that prior COVID-19 infection had a statistically significant correlation with the level of knowledge and practices (48). Researchers from the University Medical Center in Ho Chi Minh City, Vietnam explained that the history of hospitalization and hospital visit frequency had a positive association with KAP (49). Furthermore, they witnessed a significant difference in the number of COVID-19 information sources with the KAP (49). In our study, the source of information data was not collected, however, the frequency of assessing COVID-19 information was shown to be positively associated with the KAP. We argued that by contracting the COVID-19 infection, people would look for more information about the disease to overcome the condition. Hence, these two factors result in better KAP.

When a larger amount of information is presented, the ability to recall the information correctly declines. By giving the public frequent exposure to health information, better memory will be created (50). Therefore, the frequency to assess medical information will allow the patient to recall and comprehend the information properly which improved the overall KAP.

Our study provided evidence that knowledge is significantly associated with attitude but not practices, while a positive attitude is also statistically significant to promote good health measurement practices. Here, we also found fewer factors that were attributed to the practices toward COVID-19. This is probably related to the government regulation that enforced the people to obey the public restrictions, hence, fewer variations could occur in their practices, resulting in fewer components being statistically significant enough to influence them. The effective communication in medical practices developed by Ley (51) emphasized the information giver should ensure public and patient understanding of health issues by evoking their emotions. The combination of these cognitive and psychological experiences will lead to people's adherence to particular health behaviors. In addition, the information giver should be able to provide a platform that allows the public to recall the information and ensure the continuity of the cognitive–psychological circuit (51). This mechanism can then demonstrate how the KAP variables are associated with this study.

However, we also found that fewer determinant factors were attributed to the practices toward COVID-19. This is probably related to the government regulation that enforced the people to obey the public restrictions, hence, resulting in participants' practices uniformity and fewer components being statistically significant enough to influence them. This is supported by an Indonesian study conducted in West Java Province where they found that the source of COVID-19 information was strongly and significantly associated with preventive behavior. That study also discovered that their major source of information came from respected public figures (83.7%) and the local government announcement (74.7%) (52).

## Limitations of the study

This study had several limitations. First, we only collected the data using an online survey, and the survey was distributed from online platforms. The respondents of this study were then dependent on the social online networks of the distributor, in this case, the *BPJS Kesehatan*. Those who were engaging in the survey would probably also be the ones from well-developed internet networks area, with better internet literation, and with an interest in COVID-19-related topics. Thus, many of our respondents came from higher education backgrounds and had relatively higher incomes than' Indonesia's larger population. In particular, we used the convenience sampling method, where the respondents also broadcasted the survey's link to engage more respondents. Hence, the broadcast receivers may also come from a similar background to the sender, making the respondents less likely to be diverse in terms of their socioeconomic context. Moreover, this study did not cover and compare the differences between urban–rural areas since these terms cannot be categorized by their provinces alone. We then acknowledge that this study cannot fully portray the KAP toward COVID-19 in Indonesia, however, this study can serve as an insight and initial indication to conduct similar studies in the near future. Nevertheless, our study has a more balanced gender and age distribution than the previous Indonesian study where >67% of the participants were women and mainly comprised the younger population (28, 29, 53). Furthermore, this study also had respondents that were double compared to a similar study previously done by Dwipayanti et al., which also carried out an online survey to represent the Indonesian population (30).

Siddiquea et al. mentioned in their systematic review and meta-analysis that the heterogeneity of knowledge, attitude, and health practices related to COVID-19 was relatively high. This can be attributed to the different sociocultural norms in different societies (54). Hence, our study may differ if similar studies are conducted in more specific Indonesian communities or regions. However, such a study should always be developed and promoted to find the best and most distinctive community-based awareness programs, especially in multicultural societies such as Indonesia. This is particularly true because what is simple and understandable to one community might not be interpreted similarly by others.

## Conclusion

Knowledge is still attributed to the attitude, which may lead to people's practices after the delta variant wave. This study underlined that medication knowledge was the lowest compared to other knowledge aspects, which could lead to a false attitude and practices in seeking medical care. In particular, the situation during the surge of delta variants made the healthcare facilities collapse. It would not be impossible for many COVID-19-confirmed patients to seek alternatives or purchase non-scientifically proven medications, harming both their physical and financial health. The public's attitude and practices toward medication should be further studied beyond COVID-19. Moreover, this study elaborated on the impact of assessing information on COVID-19 knowledge. It should be evaluated whether the provided information was only weighted to particular themes and left the other issues behind. Hence, the public is able to comprehend the information thoroughly rather than in a scattered manner. This study also provides the determinants of the KAP, which can be further used to investigate the information distribution and the effectiveness of its communication in various groups. Therefore, the best effort can be discovered to spread the information among the less aware groups before they contract the disease. These determinants can be elaborated in any other disease study to provide more adequate evidence in promoting health KAP, not exclusively during the pandemic era but also in the more crucial post-pandemic setting.

## Data availability statement

The raw data supporting the conclusions of this article will be made available by the authors, without undue reservation.

## Ethics statement

The studies involving human participants were reviewed and approved by Faculty of Medicine, Public Health and Nursing, Universitas Gadjah Mada, Yogyakarta, Indonesia No. KE/FK/0945/EC/2021. The patients/participants provided their written informed consent to participate in this study.

## Author contributions

FH, IA, and GK: conceptualization, methodology, survey development, data management, data analysis, writing—original draft preparation, and writing—reviewing and editing. AO, ED, IT, JJ, and MR: data collection, data management, and writing—reviewing and editing. BS, CJ, DS, and WB: conceptualization, methodology, survey development, data collection, and writing—reviewing and editing. All authors contributed to the article and approved the submitted version.
